# Supporting Adult Syrian Learners With Refugee Experience in Canada: Research-Based Insights for Practitioners

**DOI:** 10.1177/00220574221091930

**Published:** 2022-05-26

**Authors:** Li-Shih Huang

**Affiliations:** 1 Department of Linguistics, University of Victoria, Victoria, BC, USA

**Keywords:** learners with refugee experience, language instruction for newcomers, Syrian adult language learners, language instruction for Syrian refugee learners, language training for Syrian learners with refugee experience

## Abstract

From November 2015 to October 2020, Canada had welcomed 44,620 Syrian refugees to more than 350 communities across the country. In 2019, it further surpassed the United States and Australia in the number of refugees settled. Lacking the necessary language skills for living and working in a new country is one of the most critical barriers refugees face. This paper aims to inform language-teaching professionals about pertinent linguistic and nonlinguistic issues as well as pedagogical implications associated with supporting adult Syrian refugee learners, drawing both on the literature more broadly and on the author’s research in the Canadian context.

## Introduction

According to the United Nations High Commission for Refugees (UNHCR, 2019), by the middle of 2020, 80 million people worldwide had been forcibly displaced. Among them, refugees accounted for 26.3 million. Syria, Venezuela, South Sudan, Afghanistan, and Myanmar constituted two thirds of the people displaced in 2019, and 40% of the 80 million were under 18. Moreover, more than 6.6 million have fled Syria since the outbreak of its civil war, making the Syrian refugee crisis the world’s largest humanitarian crisis of our time. Canada is one of the major countries endeavoring to resettle refugees. In response to this crisis, Canada had welcomed, from November 2015 to October 2020, 44,620 Syrian refugees to more than 350 communities across the country. In 2019, it further surpassed the United States and Australia in the number of refugees settled. Lacking the necessary language skills for living and working in a new country is one of the most critical barriers refugees face. This paper aims to inform language-teaching professionals about pertinent linguistic and nonlinguistic/contextual issues as well as pedagogical implications associated with supporting adult Syrian refugee learners, drawing both on the literature more broadly and on the author’s research in the Canadian context (Huang, 2021a, 2021c).

## Framing the Issues—Accessibility and Lack Alignment Of Learners’ Needs with Instruction

According to the United Nations’ Convention Relating to the Status of Refugees, the term *refugee* is defined as someone who, “owing to a well-founded fear of being persecuted for reasons of race, religion, nationality, membership of a particular social group, or political opinion, is outside the country of his nationality, and is unable to or, owing to such fear, is unwilling to avail himself of the protection of that country” (United Nations Human Rights, 1951). Canada’s prevailing approach to language-training programs—known as Language Instruction for Newcomers to Canada (LINC), funded by the Department of Immigration, Refugees and Citizenship Canada (IRCC)—is to combine refugees with immigrants. Prior to LINC’s establishment, language training in the 1950s was geared toward preparation for Canadian citizenship. Currently, federal strategy is to help newcomers (immigrants and refugees) successfully integrate into Canada by providing them with basic language training in either English or French (the official languages), as well as by supplying newcomers with knowledge about Canada, and LINC has become a key part of that program (Huang, 2021a).

As stated in the introduction, one of the most critical barriers faced by refugees is the language barrier. Still, concerns about meeting language-training needs have arisen in Canada and elsewhere, as can be seen in the flood of headlines such as “Language barriers leaves refugees facing struggle to rebuild their lives” (Summers, 2016); “‘It was very hard’: Learning English a struggle for Syrian refugees, and support not always there” (Vijayann, 2016); “B.C. has longest waiting lists for English classes in Canada” (Carman, 2016); “Edmonton refugees needing to learn English face extended wait-times” (Ryan 2016); “Two years of ups and downs for Syrian refugees who settled in Vancouver” (Waisman, 2018); and “New research shows refugees suffering from lack of English classes, despite strong public support for action by government” (Refugee Action, 2019). For adult refugee-background learners, employment is key to their resettlement and integration. According to Sam Nammoura, a refugee advocate, “the longer any newcomer has to wait to learn English, the longer they must wait to get a job and settle into their new life in Canada.” As he further noted, “to find a job, to integrate, to provide for the family, for better opportunities—English is very essential” (Klingbeil, 2016: para. 15–16).

Coupled with requests for accessible training to meet the demands, a series of interviews with Syrian learners and instructors (Huang, 2021c) revealed that instructors’ and learners’ perceptions were consistent in the sense that the language training that Syrian learners received and the language-learning needs reported by the learners did not align. This became the source of great frustration for instructors and learners’ alike. Concerns about language-learning for employment purposes were featured prominently across all data sources, as reflected by the mention of “work” and “employment” (232 and 356 by instructors and learners, respectively) (Huang, 2021a, 2021c).

A headline in the *Financial Post*, “Now we know how well Trudeau’s Syrian refugees are doing. It’s not good: For government-assisted refugees, the employment rate is less than five per cent” (Watson, 2019), is in line with previous research (e.g., Jackson, 2013; Khabra, 2017). Yet literature focusing on adult Syrian refugee learners of English in Canada, and specifically within LINC, is glaringly lacking, let alone empirical research. The headlines and the lack of research underscore issues related to current refugee resettlement efforts faced by multiple stakeholders (e.g., federal agencies, LINC providers, instructors, learners, and material developers).

## Making the Case: Contextual and Linguistic Factors

### Contextual Variables

Research has shown that learning a language and attaining needed proficiency are key challenges for newcomers in gaining access to employment, pursuing further education, and integrating into the host community (Ghadi et al., 2019). As Masri and Abu-Ayyash (2020) stated: “The most important step in language acquisition is to understand the factors that lead to SLA [second language acquisition] and that lead to successful integration within a new society” (374). As noted above, refugees and immigrants are often combined when implementing language training for newcomers in Canada. Yet while they do share common experiences (e.g., using a different language, working with identity issues, adjusting to a new host country), a whole host of nonlinguistic learner variables also deserve careful attention and holistic consideration. Refugees are a complex and distinctive group of learners because of the unique circumstances they have faced, including being forced to flee or migrate, loss of family and community, interrupted schooling and work, trauma, post-traumatic stress disorders, and resettlement in a place they did not choose (Brewer, 2016; Masri & Abu-Ayyash, 2020). Arriving in the host country with this migration history is vastly different from arriving as an economic, family class, or student immigrant; consequently, their life histories or premigration backgrounds (Bigelow et al., 2020) must be considered in developing linguistically and culturally sensitive approaches to instruction.

In line with Warriner et al.’s (2019) work, the data from a recent follow-up survey of 2,242 Syrian refugee learners about their English language-learning needs (Huang, 2021d) also displayed wide variation in their prior experiences of schooling, and, by extension, their various levels of source language proficiency and *perceived* target language ability (Table 1). For example, 21.83% of the adult respondents (male: 72.91%; female: 26.78%; preferred not to say: 0.31%) lacked a high school diploma, and yet the typical instructional approach tends to assume that learners are proficient in literacy skills in their own language. Most learning activities also assume or focus on literacy-based tasks.

**Table 1.** Syrian Refugee-Background Learners’ Reported Proficiency in the Four Language Domains.

Language domain	LINC stage I (CLB 1–4)	LINC stage II (CLB 5–8)	LINC stage III (CLB 9–12)
Speaking	50.50	46.68	2.82
Listening	47.69	47.89	4.43
Reading	36.52	55.74	7.75
Writing	53.02	42.86	4.12

*Note.* Percentages may not sum to 100 because of rounding. CLB (Canadian Language Benchmarks) stage I (CLB 1–4): Initial Basic Ability; CLB stage II (CLB 5–8): Fluent Intermediate Ability; CLB stage III (CLB 9–12): Developing Advanced Ability.

While studies are lacking in the Canadian context, several studies in other learning or geographical contexts have pointed out the complex, interrelated challenges and barriers refugee-background learners face (e.g., Benseman, 2014; Bigelow et al., 2017; Hadfield et al., 2017; King et al., 2017). Our work echoed these studies in revealing limited or interrupted formal schooling experiences, experiences of displacement and trauma, loss of family and community, issues with psychological well-being, decreased capacity to focus and retain information dealing with abstractions, difficulties with learning how to learn in formal educational settings and with understanding the norms of social or learning behaviors in virtual or face-to-face classrooms, and limited digital literacy. All these challenges require significant adjustments by learners to the learning process. For low-literacy learners, the challenges are compounded. As well noted by Chapman and Williams (2015: 37):

Assessing complex information can present a greater challenge for low literacy background ESL learners, who often come from small or isolated communities with very different sociocultural contexts and lack of access to technology. They need to not only adjust to a new language and the cultural values of living and working through that language, but must also adjust to living in literacy-saturated, technologically oriented urban communities.

For refugee learners who have not experienced interrupted or limited formal schooling, a growing body of research has supported integrating their existing linguistic resources, including their own language and use of multimodal communication for meaning making, into their engagement with learning their target language as well as academic content (Bigelow et al., 2017; refer to Warriner et al., 2019). As pointed out by Bigelow et al. (2017), “For many refugees, connectivity is vital: smartphones and mobile technology are crucial tools for refugees worldwide” (184). The use of social media also affords opportunities for learners to develop multilingual literacies (home language[s], the target language, and digital) that reflect their communication outside of classrooms.

Canada’s refugee resettlement program, managed by the IRCC, supports most refugees under two main programs for their resettlement: the Government-Assisted Refugees Program, and the Private Sponsorship of Refugees Program. After the sponsorship program comes to an end, the immediate concern and challenge for refugee learners is securing employment to support themselves and their families financially. The lack of alignment of learners’ needs with language instruction, the mismatch between learners’ needs and employment-related language training, and the inadequacy of materials suitable for meeting learners’ needs are major limitations identified by both learners and instructors (Huang, 2021c). As one learner from our earlier survey put it, “what I need are special English courses and classes, preparing us to the Canadian Dentist Board examination. You have five years” (L019). Another said:

I come to Canada in 11 months, I study English, what your address, what your name, what’s ahh, how many have children, what name your children, how old are your son, where is this work? Not work, it’s not really for test or work related. (L013)

As yet another learner noted, “قطع السيارات كلياتها قطع السيارات لازم احفظها شو هي بالانجليزي . . . I need to remember all the technical words for trucks, the inside of the truck.” (L006; Huang, 2021a, p. x).

A report by Martani (2020) noted, “All refugees resettled in Canada . . . face early integration challenges, starting with language: the wait for language instruction is long, it is not job-specific and is not suitable for people who have low levels of education” (para 4). Khabra’s (2017) study, which examined Syrian refugees’ employment experiences in one major destination community for refugees and immigrants to Canada, concluded that “insufficient English skills, lack of Canadian work experience, poor mental health, and a limited understanding of the Canadian labour market” are among the greatest barriers identified in their economic integration (iii). Our own survey (Huang, 2021c) de signed to identify the unique language-learning needs of Syrian refugees and how they relate to their integration into Canadian society and the workforce further revealed their key language-learning concerns regarding employment.

### Linguistic Factors

The relevant variables discussed in the preceding section all interact within the language-learning process. Additionally, linguistic variables may also play a role in Syrian refugees’ learning of the English language. An instructional approach that values their learners’ own languages would require instructors to be aware of potential interlinguistic transfer issues between the learners’ own languages, in this case, Arabic, and the target language, English. In broad terms, language transfer refers to the positive and negative influences of the learners’ first language, or any other previously acquired language, on the learning of the second or target language (Odlin, 2006). Positive transfer can facilitate acquisition or use of the target language, whereas negative transfer can interfere with such acquisition or use.

While difficulties in target language learning may not arise solely or mainly because of differences between the target language and the learners’ own language(s), and linguistic differences may not always contribute to the difficulties, few instructors would deny that there is value in what learners’ first languages contribute to teaching. The effect is often manifested in target language production that deviates from the target language syntactically, lexically, morphologically, phonologically, and in the mechanics (in writing). Instructors thus naturally stand to benefit from information that would help them understand and anticipate linguistic variations that may arise in learners’ linguistic production. Instruction must begin where learners are (Huang, 2017). One way to do so where learners will be most receptive to the instruction is to understand where they are coming from and acknowledge their linguistic choices. As they make progress, the role played by their own languages will also tend to diminish (refer to Bardovi-Harlig & Sprouse, 2018).

Numerous variations of the Arabic language are spoken in Syria. Arabic is the official language, and Modern Standard Arabic is used in the education system and in printed materials, media, and official documents. Different spoken dialects are used by Syrians in their daily lives. The following paragraphs, with illustrative excerpts, summarize selected linguistic differences gleaned from oral production data (Huang, 2021c) gathered from Syrian learners at the Initial Basic Ability stage (Canadian Language Benchmarks Levels 1 to 4—that is, the *basic user* level in the Common European Framework of Reference that aims to inform pedagogy for preliteracy level learners; refer to Marrapodi, 2013). The Canadian Language Benchmarks (CLB) standard (2019) is a descriptive scale of language ability in English as a second language written as 12 benchmarks along a continuum of progression from basic and intermediate to advanced abilities. It is important to bear in mind that the goal is to raise educators’ awareness of the linguistic differences observed in the data; instructional strategies for cross-linguistic awareness instruction is not the focus of the study, but learners’ own language use is one of the major themes that emerged from the data (Huang, 2021c). The coverage here aims to prompt instructors to re-evaluate their practices. Awareness of learners’ own languages is the first step toward the possibility of adopting/adapting any strategies recommended in the literature to suit one’s instructional contexts. Some of the excerpts touch on multiple points listed below; selected examples are underlined for illustration purposes.

1. *Lexical choices:* A recent study (Huang, 2021c, 2021e) examining the oral production data of nine Syrian learners at the Initial Basic Ability stage revealed that, among the 1,257 errors coded, word choice (e.g., 1a-1f) ranked highest among the top-10 deviations related to determiners, tenses, prepositions, missing verbs, references, and pluralization. Further, the lexical profile calculated using VocabProfile showed that, excluding off-list words (8.29%) that were mainly verbal fillers, 87% of the words used by participants were in K1 (1–1000) words and 3.45% were at the K2 (1001–2000) level, with an overall type-token ratio of 0.12. For learners at the Initial Basic Ability stage, acquisition of the most frequent 3000 word families is an important threshold (Schmitt & Schmitt 2012). Earlier research has suggested that Arabic first-language users tend to experience greater difficulty than other English language learners of equivalent proficiency with reading and word recognition tasks. Various factors have been identified as sources of this difficulty (e.g., different skills required in coding English, and linguistic, cognitive, and cultural backgrounds; see Roche & Harrington, 2013).
  Excerpts:
  1a. “I will just *list* at [name] college.”
  1b. “I *get* a course for hair styling when I came to Canada.”
  1c. “My husband, he is assistant engineer in Syria. He is a *deliver, delivery*.”
  1d. “I hope in future I continue . . . my study or *another side*, another, I don’t know, *section* maybe.”
  1f. “Because you always have *empty* time, you have to fill up with something, you should not keep like being in work or study or something.”

2. *Plurals, countable and uncountable nouns:* In English (see Ahammad, 2017; Wallwork, 2012), nouns can be singular or plural (e.g., 7h, 9a, 9c), whereas in Arabic, nouns can be singular, dual (referring to two people or objects), or plural. Types of nouns in Arabic are considered in terms of gender (masculine or feminine), state (definite or indefinite), number (singular, dual, or plural), and case (nominative: e.g., the subject of a sentence; accusative: the object of a verb; and genitive: the object of a preposition; Alhammad, 2017). Some common grammatically singular uncountable nouns in English are plural in Arabic, so instances of adding -s to nouns (e.g., feedback, advice, information, equipment, money, stuff, staff, and housework) is prevalent.
  Excerpts:
  2a. “I like to make *dissert*.”
  2b. “I get a job . . . I work with hair salon for three *month*.”
  2c. “There are *people* who is famous . . . they hasn’t . . .”
  2d. “I have four daughters and two *son*.”

3. *Definite and indefinite articles:* The use of definite and indefinite articles in English is challenging for Arabic-speaking learners of English. The definite article in Arabic is less restrictive than “the” in English, a usage that tends to lead learners to overgeneralize or overuse “the” in English. The absence of indefinite articles in Arabic tends to be evident in overuse or omission of “a” or “an” with plural nouns (e.g., 3a-3e, and 6b).
  Excerpts:
  3a. “Damascus is *oldest* capital in the world.”
  3b. “I was learning in *the* Camosun College.”
  3c. “It’s nice to have something to do . . . new thing to do in *the* life.”
  3d. “We can find *a* work.”
  3e. “I just study [English] in *the* school.”

4. *Prepositions and phrasal verbs:* Prepositions and phrasal verbs can be areas of great difficulty for learners at the Initial Basic Ability stage. The number of Arabic prepositions has been a matter of debate, but no one in the literature has argued against the complexity of the system of Arabic prepositions in its functions, classifications, and construction (see Husn & Zaher, 2020). The misuse of English prepositions is often seen in unnecessary insertions (e.g., “in inside”), omissions, and incorrect uses/substitutions (e.g., 1a, 1f, 4a-fi, 6b, 7g, 8c). Certain prepositions in Arabic have different semantic meanings and functions that can interfere with learners’ selection of the English equivalents. As for phrasal verbs, opinions also vary regarding their existence in Arabic (see Gandorah, 2015). The challenges lie in their frequency (how common they are), construction (phrasal verbs word order), meaning (polysemy and semantic opacity), and usage (context) (Martinez, 2013). Avoiding production and misinterpreting perceptions are common phenomena.
  Excerpts:
  4a. “The famous dish my country like rice with chicken.”
  4b. “I think I will *drop off* [name of the LINC provider] . . .”
  4c. “To Canada, I just know some alphabets, but I work *for* writing. Now I am good.”
  4d. “I work with my husband factory of masks. We make masks.”
  4e. “I move to here *before* four month.”
  4f. “I like to listen *for* English and speak English, more than write or reading.”
  4g. “Wednesday I don’t work afternoon.”
  4h. “I came to Canada *for* two years.”

5. *Adjectives:* In Arabic, adjectives follow the nouns (e.g., 5a, 5b, 6b, 6d, and 7c) and must match the nouns in gender (i.e., masculine or feminine), number (i.e., singular, dual, or plural), definiteness (i.e., marked by a prefix for both the noun and the adjective; cf. English is marked by the selection of determiners), and grammatical case (i.e., case endings to mark the grammatical functions).
  Excerpts:
  5a. “First thing, in Victoria, people *very kind*”
  5b. “The park, the garden, the river *very much beautiful*.”

6. *Verb tenses:* As with other linguistic characteristics, the verb system in tense and aspect has also been an issue of great debate. Recent corpus-based research has challenged the observations or generalizations of previous studies (see Alasmari, 2018). For learners at the Initial Basic Ability stage, tense-related challenges may include tense substitution (the simple present rather than other verb aspects, e.g., progressive, perfect, and perfect progressive), tense marker omission (e.g., omission of the auxiliary verb in constructing the present perfect), substitution (e.g., what does . . . -ing?), and tense agreement (e.g., 3e, 6a-6f, and 9c).
  Excerpts:
  6a. “I *live* in Dahr. I *get* married. I *am live* in the village. Then I *get* married to live in the city . . . I *leave* Syria to Jordan. Then I *come* to Canada.”
  6b. “I *don’t work* in Syria because I *was study* in Syria . . . I was a study high school, and my dad, he had big factories cheese, and he pay for me.”
  6c. “I will live Victoria, Canada four years ago.”
  6d. “I *am walking* the Quadra Street, to Hillside, and go back my house.” (In response to: “Where do you like to go walking?)
  6e. “Last time I *will drive* 3000 km.”
  6f. “I *will study* English 5 to 6 months every day. After Covid-19, I don’t study English.” (In response to: “Do you study English now?”)

7. *Copula and auxiliary verbs:* Using Brown and Miller’s (2013) definition, a copula (*be*) is “a verb which has no content, but simply links two words or phrases” (112). Previous (e.g., Noor, 1996; Sabbah, 2015) research on learners’ written production has suggested that Arabic learners of English tend to omit the copula in their production and has also questioned the learnability of copula by students. More recently, Steiner’s work (2019) has provided a more nuanced understanding of copula omission and errors by Arabic speakers learning English at both high and low target language proficiency. As they produce English copulas, learners can access the copular structure in Arabic, but they must “learn to produce it in environments [in relation to tense, verb type, and number agreement] that are different from their first language” (2). Other errors include, for example, the omission of -*ing*, auxiliary redundancy (“are will be”), and doubling of the copula in interrogatives.
  Excerpts:
  7a. “Before, I young, I happy for play soccer but now, no soccer.”
  7b. “I *am stay* at home with my son.”
  7c. “I *am live* in Canada one years. I have three daughter, one son. My son big.”
  7d. “We *didn’t* more time to make another activities.”
  7e. “When I go to the ocean in the summer, I *can swimming*, and the winter you can go to the pool.”
  7f. “I *am like* cooking very much . . . anything cook.”
  7g. “I *am love* learning English ... I *am like* to read the story, book.”
  7h. “I have a nice guys who *is work* with me, and I have a nice boss . . . and *is work* with me.”

8. *Word order:* In Modern Standard written Arabic, VSO is the unmarked or dominant word-order pattern, whereas other patterns or variations (VOS, OVS, and SVO) are constructed to serve other pragmatic or rhetorical purposes when the structures are examined in context (Peled, 2009). Instructors might observe in learners’ oral production the deviated form of the obligatory inversion of subjects.
  Excerpts:
  8a. “You know *who is he*.”
  8b. “When the class will begin?”
  8c. “How I can help you?”
  8d. “I learn how *can I make* an appointment with doctor ... how can I reservation in a restaurant or in hotel.”

9. *Agreement:* At the Initial Basic Ability stage, learners might use numbers without using the proper form to reflect plural nouns. This practice may further connect to the use of verbs to agree with or match the preceding nouns. In English, a noun is a word that refers to an object, person, place, event, quality, idea, or action and can be singular or plural. In Arabic, as noted, nouns can be singular, dual (referring to two people or objects), or plural (Ahammad, 2017), and are either masculine or feminine; any accompanying adjectives must also agree with them (masculine or feminine). Finally, unlike English, the relative pronoun in Arabic agrees with its antecedent in number, gender, and case, and it also appears only with definite antecedents. The use of “who,” “that,” and “which” can be challenging for learners because, in Arabic, there is no differentiation between relative pronouns with human (“who”) and non-human (“that” or “which”) references (Sabbah, 2015).
  Excerpts:
  9a. “She *teach* me a lot. Last lesson she give me about the bank.”
  9b. “First daughter *live* in Holland. Second daughter *live* in Denmark. Third live in . . . Canada. I miss my daughter much.”
  9c. “I realize the same time, speaking, I couldn’t had a new vocabulary. This is *which* I miss from them, when I am studying.”

Other identified differences not covered here include overuse of the coordinating conjunction (“and”; Al-Khresheh, 2011), punctuation (no capitalization or commas), text orientation (from right to left, though numbers are written from left to right), spelling (e.g., insertion, substitution, or omission of letters), pronunciation (e.g., /b/ and /p/ [e.g., park and bark], /f/ and /v/, and /I/ and /e/; silent letters; vowel insertions in consonant clusters [e.g., “months”; and word stress]), and coherence and rhetorical issues (e.g., redundancy; refer to Ababneh, 2018; Masri & Abu-Ayyash, 2020; Sabbah, 2015; Saigh & Schmitt, 2012; Salim, 2013; Swan & Smith 1995).

LINC learners from this population are distinct, and the impact of their migration histories may variously manifest in their learning. Because the sociocultural, personal, and pedagogical factors explored here are intricately connected to the linguistic barriers refugee learners face in learning English, rigorous research must critically evaluate shifting away from the prevailing “one-size-fits-all” language-training approach that combines refugees with newcomers.

## Pedagogical Implications—Considerations and Reflection on the Field-Testing

Many instructors have found themselves unable to respond to the language, learning, and social needs of their refugee learners (e.g., Huang, 2021c; MacNevin, 2012). The following interrelated suggestions informed by theory and research are consequently offered to help instructors who support adult refugee learners as they learn English. An example from the SLEEC (Syrians Learning English for Employment in Canada) program (www.sleec-uvic.com) is provided to illustrate each consideration.

### Use an Asset-Based Approach

An asset-based approach “seeks to include rather than exclude diverse language backgrounds in classroom settings” (MacSwan, 2018: 7). As Warriner et al. (2019) noted, “valuing the resources, repertoires, and communicative assets that refugee students bring to the classroom” is critically important. Further, the approach “encourages teachers to help language users of various backgrounds understand that different language and communication practices are appropriate or useful in different situations—influenced by goals, audience, situation, and the larger (historical, ideological, institutional, social) context” (3).

Currently, the rhetoric is still ahead of practice. Although the difference-as-resource approach is nothing new, the prevailing instructional approach remains difference-as-deficit. As Rummens and Dei (2010) stated, “We need to unfailingly recognize the inherent potential of each learner and ever strive to see this potential fully realized” (n.p.). This approach highlights the need to leverage learners’ existing linguistic and cultural knowledge and focus on what they do know, not on what they lack (e.g., Lopez, 2017; MacSwan, 2018). Refugees have many “funds of knowledge” (Moll et al., 1992) and strengths, and they bring their lived experiences and identities to the learning process. Learners “without print literacy have a range of attributes that may better characterize them than one quality that they lack (Bigelow & Vinogradov 2011: 121). As Warriner et al. (2019) stated, researchers and teachers should “value and embrace refugee-background learners’ existing linguistic resources and language competencies” (32). An asset-based approach, for example, treats the learner’s own language as “critical resource(s) to be affirmed, valued, and fully utilized” (MacSwan, 2018: 2).

*From the field-testing*: The careful needs analysis conducted before the design of the program and during the implementation of the program through pre-task design focused on activating learners’ background knowledge and using it as the basis to scaffold their performance of the main task as one of the key design principles central to lesson design across levels. For example, a story-telling (titled *Stories of My Life*) lesson that connects to speaking in personal and professional settings taps into events from individual learners’ past, present, and future, using objects or pictures (that do not require print literacy) shared by learners that represent something meaningful to them—a representation of something that they are proud of (past), that is important to them (present), and that they aspire to (future). The process draws on individual learners’ experiences, existing world and linguistic knowledge, and identities in the learning process. For a full description of the lesson design, the theoretical basis, and its detailed implementation procedures, refer to Huang (in press).

### Afford Opportunities to Navigate Identity Issues

Instructors need to consider providing room for learners to explore their unique identities, which are in flux, and to navigate the contradictory labels for these identities (e.g., being professionals in their home countries but needing to seek temporary employment and recertification in order to re-establish a professional and social identity) as they go through resettlement. As Rummens and Dei (2010) cautioned: “Identification processes—particularly for contested identities—affect learning, and thereby the educational performance and associated life outcomes” (n.p.).

*From the field-testing*: The *Stories of My Life* lesson (Huang, in press) prompted rich sharing of learners’ accomplishments (including being a business owner, working as a pharmacist, operating a multinational corporation, and being a proud caregiver). Their current struggles and resilience in their work to achieve their dreams and aspirations are a clear examples of lesson designs that afford learners the space to explore their identities in flux. For example, for one learner, the proudest moment was represented by a picture of him sitting at a large desk on the day he set up his own company before moving to Canada. He then showed the image of a door of his first house in Canada, which he said “had changed his life forever,” and his calendar was an object that represented his dreams for the future. Proudly, he showed a calendar that contains his detailed plan from 2020 to 2025 to achieve his professional aspirations. Giving learners the space to voice and validate their stories, stories that are intricately connected to the now, and their learning process is essential.

### Align Needs with Instruction

Recent needs assessment research has revealed a mismatch between learners’ perceived needs and their perceptions of the instruction received from both the learners’ and instructors’ perspectives (Huang, 2021c). Navigating the language program’s instructional and assessment demands, external proficiency standards (for studies, work, citizenship, or professional certification), and learners’ needs is an ongoing challenge. Lessons can, however, be infused with personal relevance by factoring in learners’ needs in order to create engagement and meaningfulness.

*From the field-testing*: Even with lessons at various levels tailored to learners, using a wide range of positions based on employment statistics and learners’ reported data about employment, it is never possible to cover all jobs. For each lesson, acknowledging that securing a job with a specific set of communication skills that may be experimented with is not the intended goal. Highlighting ways that the skills are transferable and relevant to their development of communication skills for their intended work is critical to making each lesson meaningful. For example, becoming a supermarket cashier might not a position that learners aspire to, but the lesson creates a communicative context for learners to practice making small talk, asking questions for clarification, and dealing with challenging customers. These are skills that are transferable beyond the supermarket context.

### Strengthen Language Learning for Employment Opportunities

The common thread connecting adult learners (Huang, 2021c, 2021d) was their need to learn English for employment opportunities—one group of learners with limited literacy and education, and the other with professional credentials prior to arrival in the host country. This urgent need was reflected in the mention of employment or work nearly 600 times within a subgroup of 17 learners and instructors. The fact that over 2,200 Syrians responded to the Syrians Learning English for Employment in Canada program underscores the need to strengthen the development and implementation of English for employment opportunities (e.g., Huang, 2021c; 2021d 2020, 2021b; Ghadi et al., 2019; Sturm et al., 2018).

*From the field-testing*: Various jobs across industries have been identified and used as the basis to develop over 21 units across various roles (e.g., cashiers, delivery drivers, stock clerks, restaurant bussers, restaurant servers, medical assistants, entrepreneurs, teachers) and different contexts or scenarios (e.g., handling job interviews, dealing with challenging colleagues or customers). This process follows the task-based instructional framework (Long, 2015; WIllis & Willis, 2007), that is the pre-task (for priming), main-task (the real-world task), post-task (for focusing on forms), and follow-up task (for consolidating learning and promoting transferability) cycles (see Huang, in press). The task is engaging (needs based), and is primarily focusing on meaning for the main task cycle, with a clear, measurable outcome (e.g., to carry out an exchange relating to a real-world task). There is also a post-task form-focused cycle that aims to help learners make sense of the language and strategies they have just experienced in context and highlights what they will likely encounter again in the real-world. This follow-up task or homework serves to consolidate and create engagement.

### Connect to the Outside

The need to connect instruction to everyday life (Condelli et al., 2009) through task design and community-integrated learning is supported by research showing the cognitive, affective, social, and cultural benefits of inclusion (O’Connor, 2012). Exploring potential partnerships with the community in suitable areas or areas that match learners’ needs (e.g., community language and cultural activities, social service agencies, schools) could not only develop linguistic skills and cultural knowledge, but also foster social connections between refugee learners and their local community (see Clifford & Reisinger, 2019).

*From the field-testing*: Emerging from the needs assessment data was the theme of community-integrated learning. While the field-testing of the program did not involve a partnership with the community due to Covid-19 and the delivery of the program only in remote learning mode, SLEEC talk, a monthly gathering involving learners and teachers in game-based activities that promote cultural and language exchange was one way of fostering social connections among learners and a sense of community. This is one area with promising potential that deserves program administrators’ consideration.

### Value Multimodal Pedagogies

**Figure 1.**
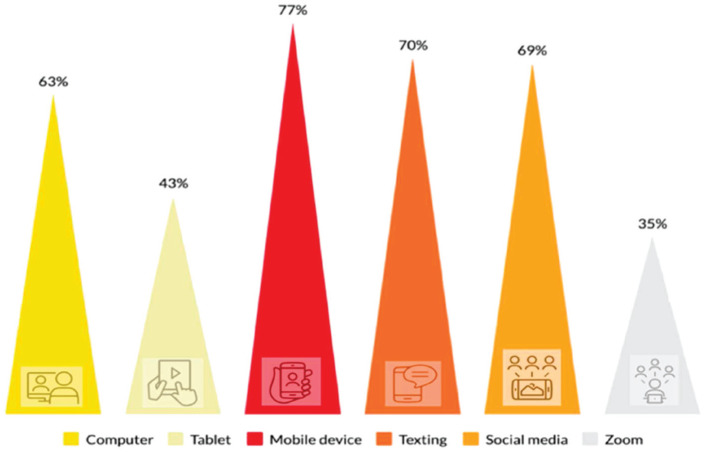
Syrian Refugee-Background Learners Reported Technology Use as “Often”.

Moving beyond a text-centered approach is important for refugee learners whose learning may be affected by the wide-ranging linguistic and nonlinguistic variables covered in this paper. While a previous study showed that computer literacy for learners at the Initial Basic Ability stage required mediation for them to benefit from its use (Huang, 2021c), most learners identified themselves as regular users of various technological tools (see Figure 1). Specifically, Syrian refugee-background learners reported using such tools “sometimes” and “often” (computers: 87.59%; tablets: 70.58%; mobile devices: 86.38%; texting apps: 85.35%; social media platforms: 86.37%; video conferencing platforms: 66.63%; see Table 2).

**Table 2.** Syrian Refugee-Background Learners’ Reported Technology Use.

	Frequency of use				
Tools	Never	Seldom	Sometimes	Often	N/A
Computer	3.97	3.76	25.03	62.56	4.68
Tablet	15.46	9.16	28.28	42.73	4.37
Mobile device	1.32	2.64	9.46	76.91	9.66
Email	3.66	4.27	18.41	67.24	6.41
Texting (e.g., Whatsapp, Face time, Messenger, Line, etc.)	3.26	3.97	15.16	70.19	7.43
Social media (e.g., Facebook, Twitter, Instagram, YouTube)	2.24	3.87	17.40	68.97	7.53
Video conferencing (e.g., Zoom, Google Classroom)	17.70	10.99	31.74	34.89	4.68

*Note.* *N* = 983. Percentages may not sum to 100 because of rounding.

Empirical studies (see Bigelow et al., 2017; Mccaffrey & Taha, 2019) have shown that embracing multimodal pedagogies may better help learners deal with real-life situations stemming from their migration experience or their current integration challenges. The pedagogical approach originated by the New London Group (1996) stresses the idea of a pedagogy of multiliteracies, which includes “modes of representation much broader than language alone” (64); it also promotes providing “real opportunities for students to express their individual cultural experiences built on their linguistic resources” (69) and constructing meanings through diverse modes of representation (e.g., previously learned languages, images, sounds, gestures, and tools; Laadem & Mallahi, 2019; Reyes-Torres & Rage, 2020). The approach acknowledges the multidimensional nature of literacy and a process of learning that involves the cognitive, conceptual, sociocultural, and affective dimensions.

*From the field-testing*: Based on the needs assessment, the use of the YouTube platform has been integrated into the instruction in the form of the follow-up task for consolidation of learning and generative language use, following the *Review, Reflect, and Create* sequence. The Review clip (labeled Level 1 Challenge) contains the lesson covered in a unit; the Reflect clip (labeled Level 2 Challenge) embedded individual learners’ own oral production recorded before the lesson and after the form-focused segment in the post-task, done for reflection. Depending on the nature of the task, learners might be asked to transcribe their recording first before answering the question: *What improvements have you noticed? What would you say differently next time?* Engaging in critical reflection is key to promoting self-regulated learning (Huang, 2021b). Here below is an excerpt that illustrates the reflection component for a unit on small talk in the professional setting (titled *How’s Your Work Going?)* that is integral to the follow-up task cycle.

In the first recording, it was difficult for me to start talking, so it was starting from X. It was also difficult for me to keep the conversation going. I was waiting for the other party to finish talking, and I didn’t know what to say at the end. Whereas in the second recording I was able to somewhat start the conversation and keep it going and I also managed to finish it well I think ^_^. (Omar, Group J, June 8, 2021)

All clips can only be accessed by the individual learners. Within the field-testing phase (five months), the private clips were accessed by 30 learners over 2,500 times, which provided a measure of engagement in learning by students mediated by YouTube clips. Other open-access tools utilized in the instruction included Kahoot! (for purposes of priming or consolidation of learning), Quizlet (for vocabulary building and consolidation purposes), Jamboard (for various task types: listing, ordering/sequencing/sorting, matching, and comparing) (Willis & Willis 2017), Padlet (for personal experience sharing), Google slides (for form-focused work), and audio-/video-clips (for various pedagogical purposes: priming, promoting noticing, form-focused work, and consolidation).

### Harness the Power of Learners’ Own Language

A growing body of research across various age groups has shown the benefits of using a learner’s own language(s) (e.g., for clarifying concepts or providing instructions during teaching, lowering cognitive load, building relationships, and acknowledging learners’ cultural and linguistic identities) to promote target language development (e.g., Condelli et al., 2009) and develop multilingual and multimodal pedagogies (see Blair et al., 2019; Warriner et al., 2019). The brief coverage of linguistic differences given here illustrates some of the linguistic-related factors that may play a role in a learner’s language production manifested in different ways (errors, avoidance, overgeneralization, overproduction, and constraints on learners’ hypotheses and choices). Being armed with knowledge of their learners’ first language can help instructors acknowledge the equal validity of that language and become aware of potentially linguistic-related sources that may contribute to their learners’ choices; additionally, it can help them explore ways to address differences by drawing on that language. Rather than conforming to the prevalent English-only policy without discrimination, it is better to explore ways for refugee-background learners at the Initial Basic Ability stage to value their own language as an asset on which to build their target language literacy. While teaching the target language, instructors can leverage learners’ existing linguistic and strategic resources and draw on their knowledge of interlinguistic transfers to inform their teaching.

*From the field-testing*: Ultimately, it is up to individual instructors to navigate own-language use on their own terms in light of their personal experiences, teaching contexts, institutional requirements, teacher/learner preferences, and research findings. Within the program, clarifying how learners’ own language use is viewed as a mediational tool for learning, shifting learners’ perspectives about the value of speaking Arabic, respecting learners’ identities, needs, and preferences, and celebrating their bilingual capability are reinforced across levels. Recognizing that learners’ own language can play a role in their communication, through an understanding of the linguistic factors covered in this paper, is the first step in helping them to make informed linguistic and rhetorical choices. Observe why and how learners use their own languages—for maintaining social relationships, for mediating learning, for task management (Huang, 2017)? A growing body of literature has offered instructional strategies for promotion of translanguaging repertoires as resources and strategies for cross-linguistic transfer or awareness instruction (e.g., Ballinger et al., 2017; Tian et al., 2020; Jiménez et al., 2015). Instructors can experiment with strategies such as not prohibiting the use of Arabic or allowing dual language projects for assignments and group work, permitting the use of dictionaries, drawing attention to the linguistic differences between the target and the source languages through patterns finding, and language and cultural sharing (e.g., conversational starters, saying/a phrase of the day, cultural activities such as cooking and storytelling), and the use of own languages for planning, research, note-taking, or problem-solving and so on. Beyond cognates, contrastive linguistic analysis, comparing patterns, cross-linguistic awareness instruction, with Arabic as the first language and English as the additional language, merit empirical exploration.

### Embrace Pedagogical Openness

Although all instructors can relate to feeling the need to engage learners with tasks and activities, Wilde (2003), drawing on insights she had gained from her parenting experience in relation to teaching, reminded readers of the need for “openness to the moment” (117), for learning to “let go of self-centered concerns,” and for hearing the “truth,” which is never “a final, decontextualized phenomenon.” This kind of learning “opens up the possibility to understand” and be understood (119–120). The approach of pedagogical openness thus “requires being open to the infinite possibilities always inherent in each new cry—and in each new encounter” (122). This approach especially applies to the work of supporting learners with refugee experience where, given the best of intentions to fulfill learning needs and engage learners, a deeper engagement may arise from “develop[ing] and enlarge[ing] our capacity to interpret and appreciate the living connections between our students and ourselves” (122).

*From the field-testing*: While the program has been developed through needs assessment derived from multiple sources of data (surveys, interviews, and oral production) from both learners’ and instructors’ perspectives (Huang, 2021c), the use of a task-based instructional framework is aligned with the need for pedagogical openness in that the pre-task cycle places learners at the center of the learning prism, where the learning begins with an openness to understand and to be understood and the intention to engage learners where their needs are and to connect with learners through their experiences, linguistic or non-linguistic. Unlike the traditional, deductive approach embodied in a sequence of presentation, practice, and production, the pre-task, which draws on learners’ funds of knowledge, is designed to scaffold learners’ production of the main task, using their existing linguistic and strategic resources. Based on the process and the production (where learners’ sense of awareness about their language use is most heightened), instructors in the post-task cycle can address the needs of the learners in the most meaningful way. This approach requires both instructors and learners to embrace fluidity in the learning process, where the instruction lies in the ebb and flow of the learning in the moment and is embedded in the specific communicative context of the unit. This process naturally requires some adjustment by both instructors (in letting go of the need to maintain control and the need to tell rather than elicit in order to expand understanding and connections between learning and life experiences) and learners (in letting go the need to emulate some “model speech” or production). Individual learners’ levels of openness to the approach may vary. A span of three to four units is usually when a shift toward experimentation begins to occur.

Finally, the need to understand and accommodate learners needs to be kept in mind, as discovered throughout the field-testing phase of the program. The degree to which the experiences of refugee learners affect their learning varies individually. Navigating personal and collective experiences may be compounded by the loss of support networks and a sense of uncertainty about financial or employment circumstances, and, for many, an urgent need to reach a certain language proficiency threshold for citizenship, study, or professional certification purposes. While recommendations about trauma-informed pedagogy are available (e.g., refer to UNHCR 2019; Capstick, 2018), little research has been conducted about the LINC context in Canada. While this general approach to understanding learners applies to those of any background, it is especially important and delicate in developing instructors’ awareness of and empathy with learners who have experienced displacement, forced migration, and trauma. Experiences of separation or loss of family or community owing to political, religious, and ethnic conflicts may affect their learning and their interactions with peers, instructors, and work. Understanding their learners’ life experiences can help instructors maintain pedagogical openness (Bigelow et al., 2020) and explore ways to support their learning that are culturally, linguistically, and ethically sensitive.

## Conclusion

The plight of refugees is a major global crisis. Canada is among the English-speaking countries where displaced Syrian refugees have resettled; consequently, it is having to address enormous challenges related to language training in its efforts to resettle this unprecedented influx (e.g., Huang, 2021c; Bigelow et al., 2020; Ćatibušić et al., 2019). As Warriner et al. (2019: 7) stated:

Expanding teachers’ (and students’) views on what refugee-background students know and can do requires changing what pre- and in-service teachers learn and reflect on during professional development. A more nuanced view of who refugee-background learners are, their existing linguistic resources, and their uniquely challenging life experiences will help teachers recognize possible ways to leverage resources such as multilingualism, familiarity with multimodal practices, digital literacies, or life experience.

There is no “typical” learner with refugee experience. A greater awareness and understanding of refugee-background learners and their life experiences will enrich thinking on lesson planning, instructional approach, materials development, curriculum design, and assessment methods. Informed instructors who can tailor their instruction to the unique experiences of refugee-background learners are better positioned to foster inclusion in their classrooms, where educational policies might be slower to catch up in working toward ensuring that refugee learners do not remain a marginalized group in schools and society (Brewer, 2016). They can also better meet their learners’ needs and help them achieve their language-learning goals while acquiring the language skills they need to function in society. This support to refugees will thereby contribute to strengthening equity, diversity, and inclusion in Canadian society and beyond.
